# Survey of vulnerable Amazonian manatees using environmental DNA (eDNA): A method for survey in remote field settings

**DOI:** 10.1371/journal.pone.0339410

**Published:** 2026-02-04

**Authors:** Kaitlyn Romoser, Shizuka Hashimoto, Nasrah M. C. Hamdan, Leonardo Sena, Miriam Marmontel, Tomas Hrbek, Izeni P. Farias, Kirk O. Winemiller

**Affiliations:** 1 Department of Ecology and Conservation Biology, Texas A&M University, College Station, Texas, United States of America; 2 Laboratório de Evolução e Genética Animal, Departamento de Genética, Instituto de Ciências Biológicas, Universidade Federal do Amazonas, Manaus, Amazonas, Brazil; 3 Center for Advacnced Biodiversity Studies (CEABIO), Biological Sciences Institute, Federal University of Pará, Guamá, Belém, Pennsylvania, Brazil; 4 Instituto de Desenvolvimento Sustentável Mamirauá, Tefé AM, Brazil; 5 Department of Biology, Trinity University, San Antonio, Texas, United States of America; Niigata University of Pharmacy and Medical and Life Sciences, JAPAN

## Abstract

The only exclusively freshwater lineage of Sirenia, the Amazonian manatee *(Trichechus inunguis*) is listed by the IUCN as vulnerable, with populations projected to decline further during the coming decades. Given that illegal hunting, pollution, habitat disturbance and other impacts are ongoing, it is imperative to assess the distribution and abundance of this unique, elusive aquatic mammal. In this study, we used environmental DNA (eDNA) methods to test for *T. inunguis* presence at three locations along the longitudinal gradient of the Amazon River and its tributaries (Tefé, Manaus, Belém). At each location, water samples were collected at sites spanning a disturbance gradient from urban to protected reserves. We developed a field methodology to preserve DNA for up to 13 days or more without requiring freezing or cooling of samples. This method performed similarly to traditional cold-storage methods used for eDNA research. In the lab, DNA was extracted from the samples followed by PCR amplification, and Illumina sequencing. Detection of Amazonian manatee DNA was more than three times greater in the western Amazon (Tefé and Mamirauá Reserve) where human activity is low. Manatee DNA was detected at six sites in the central Amazon (Manaus) and in only two sites in the eastern Amazon near the coast (Belém) where human populations and impacts are greater. eDNA methodology was effective for detecting manatees and is expected to be useful for estimating their broader distribution as well as surveying other aquatic species in tropical rivers.

## Introduction

The Amazonian manatee (*Trichechus inunguis*) is the only exclusively freshwater Sirenia and smallest member of the order [1]. The species is endemic to the Amazon River and its tributaries in Brazil, Ecuador, Peru, and Colombia [2]. During the Amazon’s wet season (December to May), manatees inhabit floodplains where aquatic macrophytes are plentiful. During the dry season, most manatees seek refuge in deeper waters within river channels and lakes [3].

Amazonian manatees have few natural predators, and humans pose the greatest threat to their survival [4]. In the past, hunting posed the greatest threat to manatees [5]. From 1930 to 1973, manatees were heavily exploited for leather, fat, and meat. Hunting of Amazonian manatees was prohibited by the Brazilian government in 1973 [6]; however, this has not completely stopped hunting of manatees by indigenous people for food [7]. Currently, the principal anthropogenic impacts are habitat destruction and loss of animals due to subsistence hunting or entanglement in fishing nets [7, 4]. Changing land use in the Amazon affects aquatic habitats and food sources of manatees [8]. Pollution from agriculture or mining also can reduce aquatic vegetation that is consumed by manatees [9]. Given their sensitivity to these impacts, manatees have been proposed as sentinels for the emergence of threats to other components of aquatic ecosystems [10].

Since population assessments of Amazonian manatees began three decades ago, the species has been listed as Vulnerable by the International Union for Conservation of Nature (IUCN) Red List, with numbers predicted to decline by at least 30% within the next 75 years. Recent assessments of Amazonian manatee distribution and abundance have been inefficient and there are major data gaps [11]. Local collaborators and communities have noted fewer Amazonian manatee sightings in recent decades, especially near populated areas [7, 12]. Although visual sighting and side-scan sonar techniques have been used to detect manatees in the Amazon, these methods are less reliable due to the species’ elusive nature and difficulty of detection in dense aquatic vegetation [11]. Manatees are often only visible when at the surface to breathe, meaning observations are limited in the turbid waters often found in the Amazon. Here, we test the effectiveness of environmental DNA (eDNA) methods for detecting Amazonian manatees.

Use of non-invasive eDNA methods for detection of aquatic species has increased greatly in recent years and has been shown to perform equally or better than traditional survey methods involving nets, traps, or visual censusing. For example, in Australia, an eDNA survey of two fish species proved to be more efficient with greater sensitivity than electrofishing and net fishing [13]. In the Arabian Gulf, 73 families of vertebrates, including five marine mammal taxa were detected using eDNA [14]. In the Amazon specifically, eDNA has been used to detect species of fish and tetrapod, including endangered mammals such as, giant otters and river dolphins [15, 16]. Valsecchi et al. [17] used eDNA to detect cetacean species in the Mediterranean Sea and obtained results that corroborate or exceeded visual sightings. In Florida, West Indian manatee detection was higher using eDNA than aerial surveys [18]. Hunter et al. [18] predicted eDNA would be particularly useful in regions such as the Amazon where manatees are difficult to locate in densely vegetated habitats.

**Fig 1:**
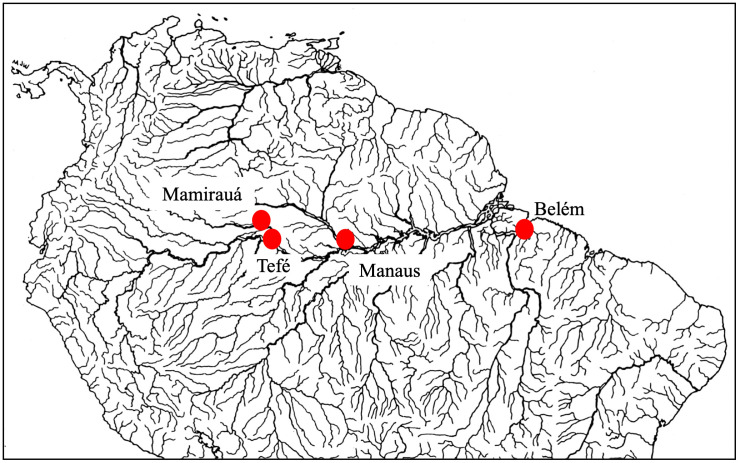
Sampling site locations. Dot symbols indicate sampling sites along the Amazon River and its tributaries. From west to east: Mamirauá Sustainable Development Reserve, Tefé, Manaus, and Belém.

In this study, we used eDNA to detect Amazonian manatees in three regions (upper, central, and lower) of the Amazon River and its tributaries (Fig 1). The upper Amazon region includes the Mamirauá Sustainable Development Reserve near the city of Tefé, an area that has relatively little human impact compared to areas near large human settlements. Because manatees have been recently observed in this area, sites near Tefé provide a good test for eDNA detection methodology. The central and lower regions were near large cities. Manaus is a city in the central Amazon with over 2.2 million people, and Belém is a city of 1.2 million people located at the confluence of Pará and Guamá rivers, tributaries in the Amazon Delta. Manatee habitat disturbance (e.g., deforestation, boat traffic, pollution, and fishing) are prevalent in these latter two regions.

The study had two main objectives. First, to determine if eDNA can be used to detect manatees in the Amazon region. DNA can persist for up to 43 hours in lotic systems, depending on environmental conditions, such as temperature, pH, and microbial activity [19]. Thus, eDNA methodology may be effective for monitoring aquatic species in the Amazon. The second objective was to test the methodology developed by Carim et al. [20] for use in remote tropical regions. This methodology does not require cooling or freezing to stabilize DNA. Traditional field DNA preservation methods used chemicals such as ethanol [21] or solutions such as cationic surfactant [22], ATL buffer [23], Longmire’s buffer [24] or other buffers [25–27]. Many methods require cooling or freezing before laboratory analysis [28], and some require analysis to begin within a few hours of collection [29]. We used desiccant beads to dry and stabilize DNA at room temperature [20].

We hypothesized that detection of manatees is correlated with distance from large human population centers. Human impacts to manatees should be greatest near urban centers, and this may restrict manatees to habitats in rural areas with low human densities. Alternatively, manatees could be present near urban centers, but with much lower incidence of detection compared to rural and protected areas.

## Materials and methods

### Collection sites

Sampling took place during the dry season within three regions along the longitudinal gradient of the Amazon River in Brazil (Fig 1). Water quality varies between locations, while water temperature remains similar; ranging from approximately 26° C and 28° C. Mamirauá Reserve is dominated by “white-water” conditions with high concentrations of suspended sediments that yield high turbidity, slightly acidic to slightly alkaline pH levels (pH 6.2–7.2) [5], and relatively high dissolved nutrient concentrations that support ecosystem productivity. Tefé is located at the mouth of Tefé Lake, a “black water” environment. Manaus is located along the Rio Negro (“black water”) and near the confluence of two water types, “black” and “white”, the former being stained with dissolved organic compounds, acidic (pH 3.8–5.5) [5], and nutrient-poor with low concentrations of suspended sediments and nutrients [30]. Water in channels flowing adjacent to Belém are considered “white”, and hydrology in the region is influenced by ocean tides but without exchanges that affect salinity (the city is located 100 km from the Atlantic Coast). Each region included 15 sites, and at each site we collected duplicate samples, one at the surface and one 60 cm sub-surface. With recommendations from local collaborators, survey sites were selected at varying distances from urban centers in a manner that spanned an obvious disturbance gradient. In total, 90 samples were collected from 45 water bodies (littoral zones of rivers or floodplain lakes).

License for water collection throughout Brazil was issued by SISBIO/ICMBio Permanent License No. 41180−2 to TH. Genetic access is registered in the Sistema Nacional de Gestão do Patrimônio Genético e do Conhecimento Tradicional Associado under SISGEN #42871, issued to IPF.

### Field surveys

At each survey location, two people collected water samples from a boat using a GeoTech GeoPump attached to a 12V battery to draw river water through a filter. Following the USDA eDNA protocol [20], Pall Labs 47-mm, 1-µm A/E glass fiber disc filters were placed in a Pall Labs 47-mm, 500-mL magnetic filter funnel. We collected water from the surface or at 60 cm sub-surface and measured the volume of water that went through the filter. Once the filter clogged, and no additional water could pass through, we poured remaining water from the funnel. Then we allowed the filter to dry in the air for about 30 seconds before removing the filter using sterile disposable forceps. The filter was then placed into a small plastic bag containing Fisher Scientific silica gel desiccant beads and the bag was labeled with a unique number. The beads absorb water from the filter and dry it completely, thereby stabilizing the eDNA at room temperature for up to two weeks [20]. Each bag was then placed in a labeled envelope. Each envelope was labeled with an identification number, date, river name, location coordinates, surface or subsurface, and volume of water filtered. To avoid contamination between samples, gloves were changed between each site, sterile forceps were used, and the funnel was rinsed with river water at each new location before filtering. Half of the samples were kept at ambient temperature until DNA extraction, and the other half were stored at 4° C within a few hours of collection.

### DNA extraction

The following pre-sequencing procedures were carried out at the Animal Evolution and Genetics Laboratory at the Federal University of Amazonas (Brazil).

In the laboratory, we cut filters in half using sterile scissors and forceps, and then stored one of the halfs in a freezer for archival material. The remaining half was cut into strips and placed into a 1.5-mL microcentrifuge tube. Once all filters were cut and placed in tubes, we used DNeasy Blood and Tissue kits (Qiagen) for DNA extractions. Manufacturer recommendations were followed with the following exceptions: 360 µL of tissue lysis ATL buffer from the kit, 20 µL of Proteinase K, and 400 µL of DNase-free water were added to produce enough liquid to fully submerge the filters. We vortexed the samples between every reagent addition. We then incubated the filters for 2 h at 56° C in a shaking incubator. Halfway through incubation, we took the tubes out, pushed the filters down into the liquid and broke the filters up slightly with a pipette tip, then replaced the tubes back in the incubator for the remaining time. Once the 2-h incubation was completed, we added AL buffer and ethanol according to the manufacturer’s instructions. After this step, the filters disintegrated into smaller pieces. To separate the liquid from the filters, we pushed the filter pieces to the bottom of the tube using sterilized blunt-ended forceps. We poured the liquid into new tubes, centrifuged those tubes for 30 s at 10,000 RPM, and transferred the liquid above the remaining filter pellet into a DNeasy Mini spin column placed within a 2-mL collection tube. Any remaining filter pieces were discarded. From this step on, DNeasy Blood and Tissue Kit protocol was followed. We used a Nanodrop to quantify the resulting elution and stored the samples at −20° C until amplification. Both field and laboratory work implemented a contamination control procedure to actively monitor and prevent contamination occurrences. To ensure contamination control, different laboratories were used for eDNA isolation, PCR preparations, and assays.

### Amplification

Amplifications were carried out with the aim of sequencing the samples using Illumina NovaSeq X platform. We developed Amazonian manatee-specific mitochondrial D-loop primers in this study to enable species-specific amplification for genetic identification and population analyses. We used D-loop sequences published by Cantanhede et al. [31] and used Primer3web to design the primers (https://primer3.ut.ee/). The primers target the hypervariable region I of the D-loop and amplify a fragment of 221 bp (including the primers). We tested the primers on DNA extracted from manatee tissue samples stored in the Universidade Federal do Amazonas (UFAM) tissue collection. The PCR products from manatee samples were sequenced, confirming positive and specific amplification of the target D-loop region, thereby validating their use for downstream genetic analyses.

**5’–GCTCCCAGACAATATATACC**TTCCACTGTAAATTCCCAACCACATGGATATTCTTCAGTCCATTTACTCCTTGATATTGCATAGCACATCACACCTCTTAATCGTCCATAGCACATCACTTGAAATCATTCTCGTCAACATGCTTATCACCTCCATTAGGCAGTCCTTGATCACCAAGCGCCGAGAAACCAACAACCCGCCCT**CATTTTGTCCCTCTTCTC – 3’**

The total volume for the PCR was 15 µL using 0.05 µL of DNA Polymerase Technology OmniTaq 3, 2 µL of DNA, 1 µL of each primer (10µM), 1.5 µL of dNTP (8µM), and 1.5 µL of 10X buffer. We ran each sample through the following reaction: 10 min at 95° C; 40 cycles of 10 s at 95° C, 30 s at 54° C, 30 s at 72° C; 7 min at 72° C; and hold at 10° C. In all PCR assays, negative controls using ultrapure water were used to ensure there was no unintended contamination among the samples. The data associated with this study are deposited in the NCBI SRA as BioProject: PRJNA1388521 (http://www.ncbi.nlm.nih.gov/bioproject/1388521).

### Second amplification and adapter attachment

A second amplification was conducted in a 96-well plate using the products from the first PCR to attach indexed Illumina adapter pairs to each sample. The indices allowed all 90 samples to be individualized, permitting pooling all samples after amplification. The unique barcode combinations can be seen in Supporting Information (S1 Table).

The PCR cycling parameters were as follows: 30 sec at 94° C; 15 cycles of 5 s at 94° C, 30 s at 55° C, 10 s at 68° C; 2 min at 68° C; and hold at 10° C.

### Library construction and speed centrifugation

After amplification and adapter PCR, samples were purified to remove remaining primers and primer dimers using magnetic carboxyl coated beads (BOMB protocol) [32], and then quantified using Qubit. The quantified samples were then equimolarly pooled, total volume was reduced by evaporation in SpeedVac, then purified using magnetic carboxyl coated beads (BOMB protocol) [32], and eluted in smaller volume than the input volume, effectively removing any remaining small DNA fragments and PCR reagents, and concentrating the sample library.

### DNA selection and preparation for sequencing

**Fig 2:**
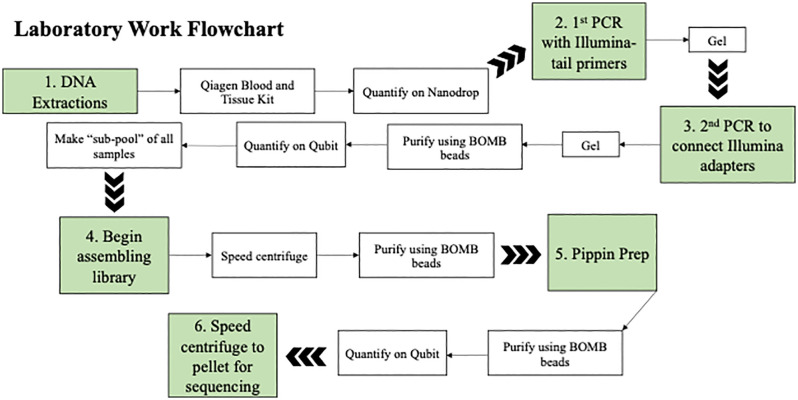
Laboratory work flowchart. Laboratory work flowchart showing detail between the main steps. Quantification determined how much DNA to add during PCR preparation or library construction.

The library was prepared for sequencing using the PippinPrep system to remove non-target products and select for fragment size. Manufacturer’s protocol was followed based on the size of the target product. Once the samples were size selected in the PippinPrep, they were purified again using BOMB to remove any PippinPrep buffers. The resulting suspension was quantified using Qubit. The library was placed in a SpeedVac system once again to dessicate it into a pellet to be sent for PE150 sequencing on the Illumina NovaSeq X platform (Novogene). Laboratory work flowchart outlining each step can be found in Fig 2.

### Data analysis

The Illumina sequencing data were demultiplex by the service provider, resulting in two paired FASTQ files for each of the 90 samples and negative controls. Each FASTQ sequence pair was filtered for the presence of the forward and reverse manatee primer using Cutadapt v4.9 [33], then merged using at least 10-bp overlap using VSEARCH v2.28.1 [34] (minimum overlap 10 bp). The resulting merged reads were dereplicated using VSEARCH and then clustered using SWARM [35]. We then used BLAST+ against a local D-loop mammalian database to classify the sequences. We considered a sequence to be an Amazonian manatee sequence if it had 98% + similarity to a *Trichechus inunguis* D-loop sequence over its entire length. We recorded the number of sequences matching these criteria in all the samples, including the negative controls. The presence of target sequence in the negative controls may be due to physical contamination during PCR steps (no visible contamination was observed during laboratory sample processing) and due to “index-jumping”, a virtual contamination. In our three negative controls, the largest number of manatee sequences we observed was 91, and therefore we considered only locations with more than 91 manatee sequences to be an indication of detection of manatees at that location.

A Chi Square test was used to compare samples in differing storage temperatures to determine if samples stored at room temperature performed similarly to samples stored at 4° C. Another Chi Square test was used to compare samples taken at the surface versus sub-surface to determine if depth affected detection probability. A final Chi Square test was used to compare rural and urban areas to determine if anthropogenic activity influenced detection probability. Using R software, logistic regression predicted positive manatee DNA samples in white-water versus black-water conditions. Logistic regression also tested the relationship between positive manatee DNA samples and the volume of water filtered. All test results refer to a p-value at a confidence interval of 95%.

## Results

**Fig 3:**
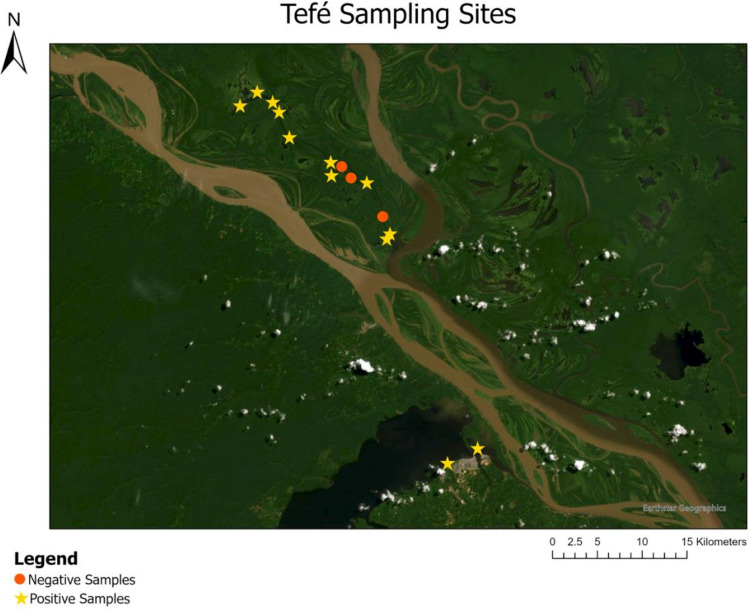
Manatee eDNA results from Tefé/Mamirauá Reserve. Positive and negative manatee DNA results from sites within the Mamirauá Sustainable Development Reserve and Tefé. A yellow star indicates that one or both duplicates returned positive. Mamirauá Reserve is located between the Japurá River to the north and the Amazon River to the south. Tefé is the town south of the Amazon River seated on the Tefé River. Each symbol represents duplicate samples, one at the water surface and another one-meter sub-surface.

**Fig 4:**
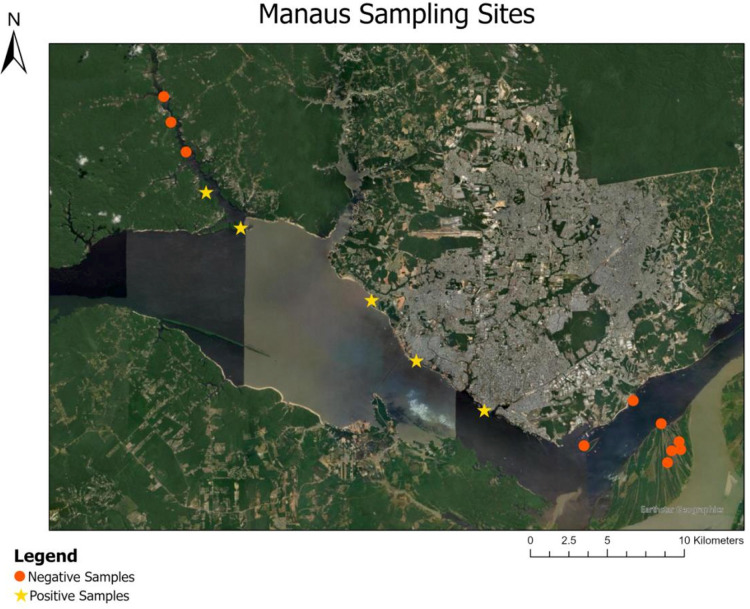
Manatee eDNA results from Manaus. Positive and negative manatee DNA results from sites near Manaus. Each symbol represents duplicate samples, one at the water surface and another one-meter sub-surface. A yellow star indicates that one or both duplicates returned positive. The Rio Negro runs along the city of Manaus before converging with the Amazon River in the east.

**Fig 5:**
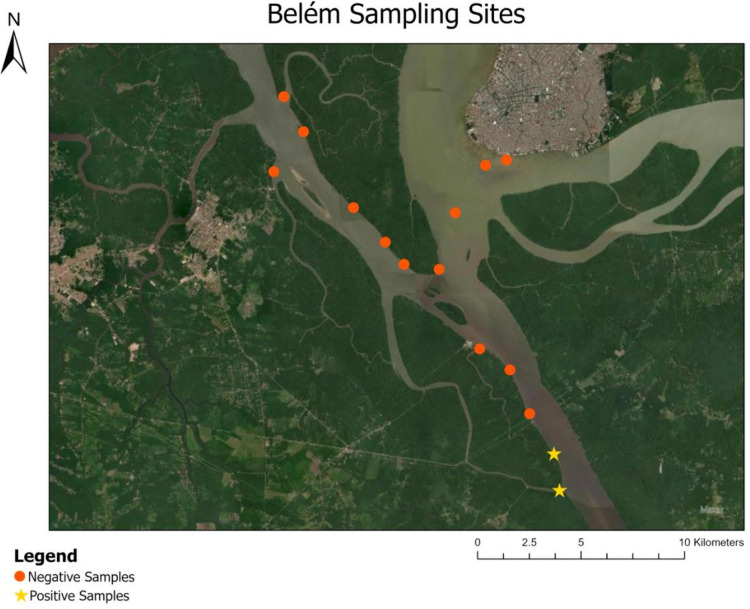
Manatee eDNA results from Belém. Positive and negative manatee DNA results from sites near Belém. Each symbol represents duplicate samples, one at the water surface and another one-meter sub-surface. A yellow star indicates that one or both duplicates returned positive. The city of Belém is bordered by Baía do Guajará to the west and Guamá River to the east. The Acará River runs southeast to northwest.

**Fig 6:**
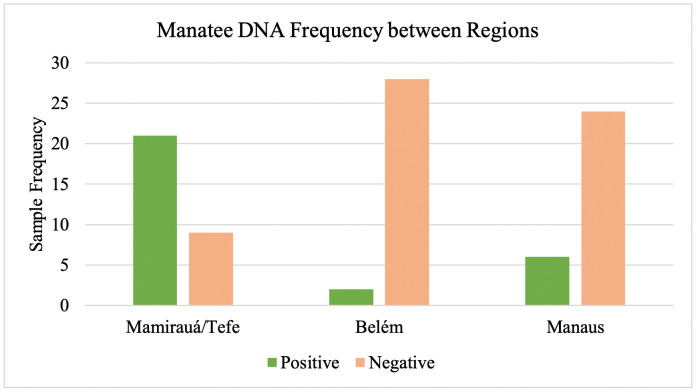
Frequency of manatee eDNA detection. Histogram comparing the frequencies of positive and negative Amazonian manatee DNA detections in the three study regions.

**Fig 7:**
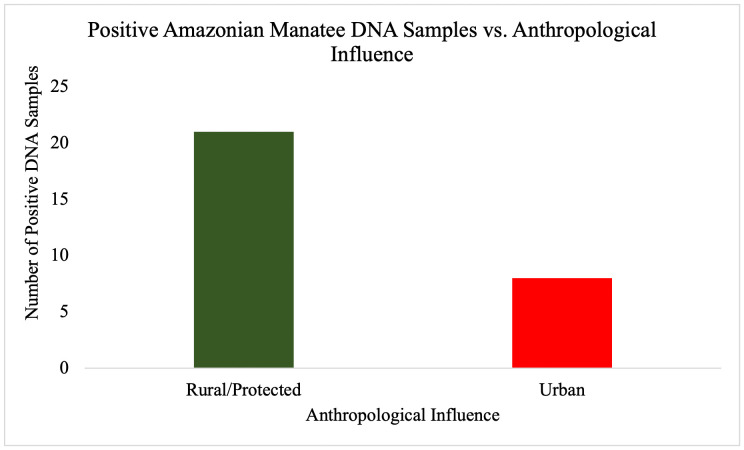
Anthropological influence on manatee eDNA detection. Manatee DNA detections in the three regions based on distance from anthropological influence. Twenty-one samples were detected in rural or protected areas and eight were detected in urban areas. Rural and urban categories were determined by overall amounts of boat traffic, settlements, and pollution in each sample area.

After performing the bioinformatic clean-up and processing analysis, the dataset for the manatee D-loop consisted of a total of 450,230 paired-end reads. To account for any background noise, the highest number of reads (91) detected in the negative controls was subtracted from the read counts in each sample. Amazonian manatee DNA was detected in 29 out of the 90 total samples collected from the three regions (Figs 3–5). Manatee detection frequencies for the three regions appear in Fig 6. Over 70% of manatee DNA detections were from within the Mamirauá Reserve and other areas distant from the cities (Fig 7). A chi square test comparing rural to urban areas did not indicate significant differences in detection based on anthropogenic influence (X^2^ = 0.25, df = 1, p = 0.619). Manatee DNA was detected in two samples in the Belém region (Fig 5) from locations outside urban areas with minimal boat traffic and relatively sparse human settlement. Two detections were obtained near the port of Tefé (Fig 3), and six detections were near the port of Manaus (Fig 4).

**Fig 8:**
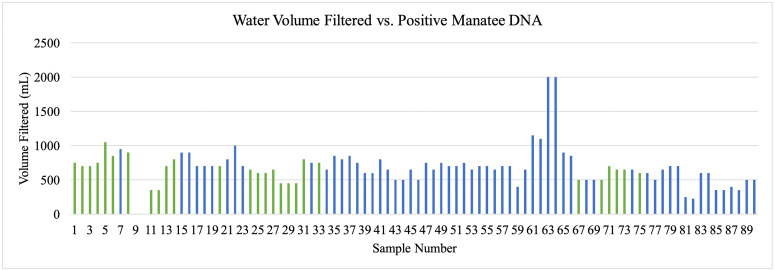
Collection depth influence on manatee eDNA detection. Manatee DNA detections based on collection depth. Fifteen positive samples were obtained at the surface, and fourteen detections were obtained at 60 cm sub-surface. There was not a statistically significant effect of water depth on manatee DNA detection.

Among the 29 positive detections, 15 were from the surface and 14 were from sub-surface (Fig 8); however, there was no statistically significant difference in detection based on water depth (X^2^ = 0.05, df = 1, p = 0.821). A chi square test comparing physicochemically distinct white versus black water did not indicate significant differences in detection probability (X^2^ = 1.18, df = 1, p = 0.276).

**Fig 9:**
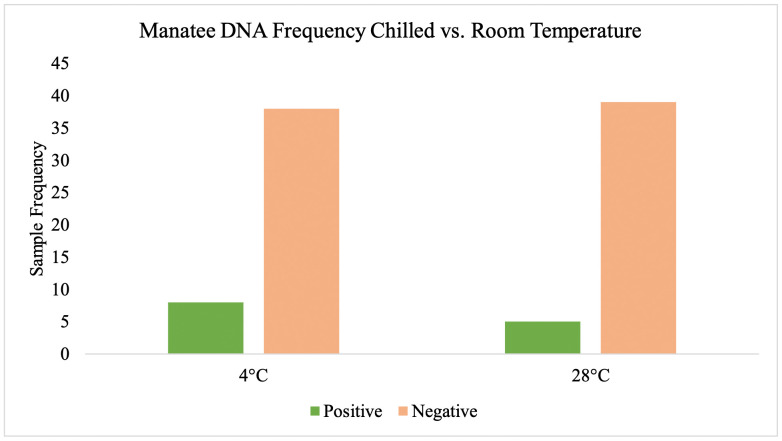
Water volume influence on manatee eDNA detection. Volume of water in milliliters (mL) filtered during collection of each sample. Positive sample volumes spanned 350 mL to 900 mL; negative samples spanned 225 mL to 2000 mL. Volumes of samples 9 and 10 were not recorded.

**Fig 10:**
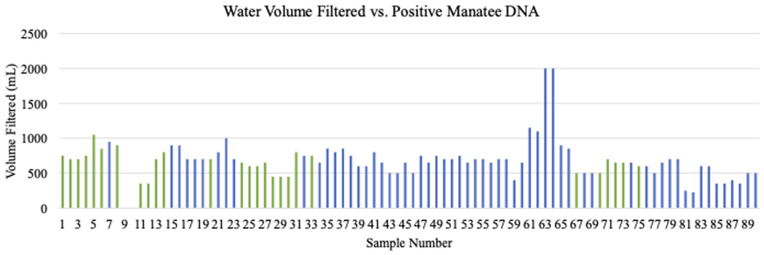
Storage temperature influence on manatee eDNA detection. Histogram comparing the frequencies of positive and negative results for Amazonian manatee DNA detection between samples stored at 4°C and 28°C. The two left bars indicate positive and negative samples stored at 4°C, and the bars on the right indicate positive and negative samples stored at room temperature. There was not a statistically significant effect of temperature on detection frequencies.

The amount of water filtered depended on the density of suspended particles that clog filters, and the volume of water filtered did not appear to influence detection of manatee DNA. Volume of filtered water ranged from 225 mL to 2000 mL, with positive DNA detections occurring between 350 mL and 900 mL (Fig 9). Logistic regression determined that there was no significant relationship between probability of manatee DNA detection and the volume of water filtered (p-value = 0.271). Additionally, there was no statistically significant effect of storage temperature on manatee DNA detection (X^2^ = 0.97, df = 1, p = 0.325) (Fig 10).

## Discussion

Surveying for Amazonian manatees involves unique challenges, and eDNA methodology adapted for remote locations in the tropics proved successful in detecting these elusive animals [20]. This to our knowledge is the first study to utilize eDNA methodology for the detection of freshwater manatee *Trichechus inunguis* in the Amazon River Basin.

Confirming our first objective, manatee DNA was detected at two or more locations within each of the three regions surveyed in this study. As expected, manatee DNA was detected more frequently within rural and protected areas, and usually was absent from samples from locations closer to urban centers. Seventy-eight percent of all manatee detections were from areas distant from urban centers. About 22% of detections were near urban areas where boat traffic was heavy. Lack of detection may be interpreted as a lack of presence, which could be due to habitat disturbance, illegal hunting, and/or inadequate food resources. It is well known that individual density is a crucial factor in the detectability of environmental DNA (eDNA); lower densities result in lower amounts of detectable DNA [18]. Due to the cryptic behavior of manatees, reliable data on their abundance and density is lacking. However, it is established that historical populations were more abundant than they are today. Furthermore, manatees are typically solitary animals, with the largest social unit generally consisting of a mother and her calf, which can remain together for over two years [5, 36, 37]. Nonetheless, temporary aggregations can occur during the reproductive and dry seasons [5]. Therefore, we cannot dismiss the possibility that density of individuals may have influenced the detectability of manatee DNA. Natural differences in water quality did not appear to influence manatee DNA detection to a significant degree, and positive results were obtained in both “black” and “white” waters. Although acidity can differ between “black” and “white” water systems of the Amazon Basin [5, 38], we hypothesize that the relatively uniform and warm water temperatures across all sampled locations [39] contributed to the consistent detection of manatee environmental DNA. Water temperature plays a crucial role in DNA degradation rates, with higher temperatures typically accelerating degradation [40]. However, given that Amazonian waters generally remain within a narrow warm temperature range year-round, degradation differences amoung sites may have been minimal, leading to no significant variation in manatee DNA detection.

Several environmental and social factors should be considered when interpreting eDNA findings. One concern of eDNA methodology is the potential for false positives and false negatives. The positive DNA sample from the port area near the center of Manaus could potentially be a false positive for the occurance of manatees. For example, eDNA from blood or other tissue could have entered the water from illegally harvested manatee meat brought to the market near the port. Although manatee hunting has been banned in Brazil since 1967, poaching has not ceased completely [1]. Manatee meat or blood leaking into the river could result in positive DNA results.

Another potential source of false positive results is varying river flow rates that affect DNA transport distance and concentration [28, 41]. In our study, the question remains if manatees were truly present near city centers and boat docks, or if organic particulates containing manatee DNA were transported downstream from another location. The waters around the port in Manaus have strong currents, which could transport eDNA in and out of the area relatively quickly. For example, the two positive DNA samples upstream of Manaus were in water with similar characteristics as the water in Belém where two positive DNA samples also appeared. It is possible that the DNA from upstream was preserved long enough to be transported to Manaus in the strong current. Additionally, there is a record of a female Amazonian manatee calf spotted in front of the city of Manaus, near Encontro das Águas in the Catalão area, in 2004 [42]. Although this sighting occurred two decades ago, we cannot dismiss the possibility that the species still exists in low densities in areas close to Manaus. Catalão is a very productive area, with large amounts of aquatic plants.

Amazonian manatee daily movement averages three km/day, but this varies depending on the season [2]. One or more manatees could have recently traveled near the urban centers, leaving behind eDNA. A study conducted in the United Kingdom reported that freshwater eel eDNA can persist in lotic systems for around 40 h under the right conditions [29], and therefore a positive detection from persistent eDNA might reveal past but not current presence of a manatee. Higher water temperature and acidity have been shown to be associated with faster nucleic acid degradation [26, 43, 44]. The Amazon River is, on average, 13° C warmer than rivers in the United Kingdom. eDNA collected in the field and held at 30° C, which is similar to the rivers sampled in this study, degrades to undetectable levels in only a few hours [45]. Thus, there is potential for false negatives when manatees are nearby, but water temperatures are warm.

Another factor that could prevent eDNA from being detected within the water samples is sedimentation after shedding from the organism. Some amount of this shed DNA sinks to the substrate where it is sequestered within sediments rather than remaining in suspension. Research conducted in rivers in Missouri, USA found that eDNA was 8–1800 times more concentrated in sediments than water [46]. Moreover, sequestered DNA can persist much longer than DNA suspended in water, which could lead to a false positive of an organism no longer in the area if the sediment is disturbed and the DNA is resuspended [47]. Additionally, species abundance contributes to overall DNA concentration [25]. Five manatees in one section of a river would naturally shed more DNA into the environment than a lone manatee. In some cases, manatee eDNA may be present in the water, but not in sufficient concentration to be amplified through PCR, leading to a false-negative result.

With regards to our second objective, eDNA can be filtered from water samples, desiccated, and preserved for two weeks or more without cooling or freezing, and stored for later laboratory analysis. This method eliminates the requirement for chemical preservation [21, 23] or cooling/freezing [48], and therefore may be a suitable option for surveys conducted in remote regions lacking access to ice or eliminating the need to transport chemicals internationally. Our findings confirm that the protocol outlined in Carim et al. [20] is effective for eDNA research in tropical environments [20]. Although this study focused on the Amazonian manatee, our eDNA methodology is broadly applicable for detecting any species that sheds DNA into its surroundings.

Findings from this study are immediately useful for ongoing efforts to conserve the Amazonian manatee, and additional eDNA surveys should be conducted in major Amazon tributaries in Brazil, Colombia, Ecuador, and Peru [2]. Future eDNA surveys of Amazonian manatees could sample during different seasons of the year and provide further insight into manatee migration patterns. Samples could also be collected from the sediment and water column to test for differential rates of detection. Greater survey effort directed at sites near populated areas could help to address the question of false positives. Finally, the desiccation method for short-term eDNA preservation should be useful for surveys conducted in remote areas in the Amazon and other regions of the world.

eDNA can serve as a conservation tool to detect elusive species, like the Amazonian manatee. Knowledge of species presence in an area can contribute to conservation regulations, protected areas, and general public education. The sampled areas near large urban centers for this study exhibit significant changes in the aquatic environment and habitat degradation. Factors such as deforestation, pollution, and bank silting contribute to the decline of manatee populations. Additionally, manatees are highly sensitive to noise, and increased boat traffic can disrupt their behavior [38]. Research indicates that many of these environmental changes are linked to proximity to urban centers, which can impact the aquatic fauna like manatee presence, prompting them to migrate to more remote areas away from urban development [11]. However, the detections we obtained close to the city of Manaus challenge this belief. We are not aware of any recent reports of sightings near the city, however, given the species secretive nature, individuals may occasionally pass nearby undetected. Use of eDNA appears to have good potential to improve accuracy of animal surveys that are a foundation of biodiversity conservation.

## Supporting information

S1 Table Unique Illumina primer pairs. Codes starting with “Q” indicate unique Illumina primers for each sample, 1–90, with negative controls, N, throughout the 96-well plate. These primer pairs uniquely identify each sample so they can all be combined during library construction and subsequent steps.
(PDF)

## References

[1] Marmontel M, Rosas F, Kendall S. The Amazonian manatee. In: Hines EM, Reynolds III JE, Aragones LV, Mignucci-Giannoni AA, Marmontel M, editors. Sirenian Conservation – issues and strategies in developing countries. University Press of Florida. 2012:47–53.

[2] Amaral R, Marmontel M, De Souza D, De Carvalho C, Valdevino G, Guterres-Pazin M, et al. Advances in the knowledge of the biology and conservation of the Amazonian manatee (Trichechus inunguis). Lat Am J Aquat Mamm. 2023;18(1):125–38. doi: 10.5597/lajam00296

[3] Arraut EM, Marmontel M, Mantovani JE, Novo EMLM, Macdonald DW, Kenward RE. The lesser of two evils: seasonal migrations of Amazonian manatees in the Western Amazon. Journal of Zoology. 2010;280(3):247–56. doi: 10.1111/j.1469-7998.2009.00655.x

[4] Marmontel M, de Souza D, Kendall S. Trichechus inunguis. 2016.

[5] Sioli H. The aquatic mammals and reptiles of the Amazon. In: The Amazon: Limnology and landscape ecology of a mighty tropical river and its basin, 56. Springer Netherlands. 1984.

[6] Domning DP. Commercial exploitation of manatees Trichechus in Brazil c. 1785–1973. Biological Conservation. 1982;22(2):101–26. doi: 10.1016/0006-3207(82)90009-x

[7] Franzini A, Castelblanco-Martinez D, Rosas F, da Silva V. What do local people know about Amazonian manatees? Traditional ecological knowledge of Trichechus inunguis in the oil province of Urucu, AM, Brazil. Journal for Nature Conservation. 2013;11(1):75–80.

[8] Brum S, Rosas‐Ribeiro P, Amaral R de S, de Souza DA, Castello L, da Silva VMF. Conservation of Amazonian aquatic mammals. Aquatic Conservation. 2021;31(5):1068–86. doi: 10.1002/aqc.3590

[9] Beck MW, Altieri A, Angelini C, Burke MC, Chen J, Chin DW, et al. Initial estuarine response to inorganic nutrient inputs from a legacy mining facility adjacent to Tampa Bay, Florida. Mar Pollut Bull. 2022;178:113598. doi: 10.1016/j.marpolbul.2022.113598 35366551

[10] Campos D de O, Souto A, Telino Júnior WR, Borges JCG, Schiel N, Alves MD de O. Behaviour and occurrence of the Antillean manatee (Trichechus manatus manatus) in relation to habitat characteristics and the influence of human activities in a protected area in north‐eastern Brazil. Aquatic Conservation. 2023;33(9):926–39. doi: 10.1002/aqc.3955

[11] Crema LC, da Silva VMF, Piedade MTF. Riverine people’s knowledge of the Vulnerable Amazonian manatee Trichechus inunguis in contrasting protected areas. Oryx. 2019;54(4):529–38. doi: 10.1017/s0030605318000686

[12] de Souza DA, Gonçalves ALS, von Muhlen EM, da Silva VMF. Estimating occupancy and detection probability of the Amazonian manatee (Trichechus inunguis), in Central Amazon, Brazil. Perspectives in Ecology and Conservation. 2021;19(3):354–61. doi: 10.1016/j.pecon.2021.03.009

[13] Piggott MP, Banks SC, Broadhurst BT, Fulton CJ, Lintermans M. Comparison of traditional and environmental DNA survey methods for detecting rare and abundant freshwater fish. Aquatic Conservation. 2020;31(1):173–84. doi: 10.1002/aqc.3474

[14] Sigsgaard EE, Torquato F, Frøslev TG, Moore ABM, Sørensen JM, Range P, et al. Using vertebrate environmental DNA from seawater in biomonitoring of marine habitats. Conserv Biol. 2020;34(3):697–710. doi: 10.1111/cobi.13437 31729081 PMC7318234

[15] Mariac C, Duponchelle F, Miranda G, Ramallo C, Wallace R, Tarifa G, et al. Unveiling biogeographical patterns of the ichthyofauna in the Tuichi basin, a biodiversity hotspot in the Bolivian Amazon, using environmental DNA. PLoS One. 2022;17(1):e0262357. doi: 10.1371/journal.pone.0262357 34982802 PMC8726463

[16] Martinelli Marín D, Lasso CA, Caballero Gaitan SJ. eDNA metabarcoding: an effective tool for vertebrate diversity studies in the Colombian Amazon and Orinoco basins. Front Ecol Evol. 2024;12. doi: 10.3389/fevo.2024.1409296

[17] Valsecchi E, Arcangeli A, Lombardi R, Boyse E, Carr IM, Galli P, et al. Ferries and Environmental DNA: Underway Sampling From Commercial Vessels Provides New Opportunities for Systematic Genetic Surveys of Marine Biodiversity. Front Mar Sci. 2021;8. doi: 10.3389/fmars.2021.704786

[18] Hunter M, Meigs-Friend G, Ferrante J, Takoukam Kamla A, Dorazio R, Keith-Diagne L, et al. Surveys of environmental DNA (eDNA): a new approach to estimate occurrence in Vulnerable manatee populations. Endang Species Res. 2018;35:101–11. doi: 10.3354/esr00880

[19] Fish J, Raule N, Attardi G. Discovery of a major D-loop replication origin reveals two modes of human mtDNA synthesis. Science. 2004;306(5704):2098–101. doi: 10.1126/science.1102077 15604407

[20] Carim K, McKelvey K, Young M, Wilcox T, Schwartz M. A protocol for collecting environmental DNA samples from streams. USDA General Technical Reports. 2016.

[21] Bressan EA, Rossi ML, Gerald LTS, Figueira A. Extraction of high-quality DNA from ethanol-preserved tropical plant tissues. BMC Res Notes. 2014;7:268. doi: 10.1186/1756-0500-7-268 24761774 PMC4005624

[22] Yamanaka H, Minamoto T, Matsuura J, Sakurai S, Tsuji S, Motozawa H, et al. A simple method for preserving environmental DNA in water samples at ambient temperature by addition of cationic surfactant. Limnology. 2016;18(2):233–41. doi: 10.1007/s10201-016-0508-5

[23] Majaneva M, Diserud OH, Eagle SHC, Boström E, Hajibabaei M, Ekrem T. Environmental DNA filtration techniques affect recovered biodiversity. Sci Rep. 2018;8(1):4682. doi: 10.1038/s41598-018-23052-8 29549344 PMC5856736

[24] Williams KE, Huyvaert KP, Piaggio AJ. No filters, no fridges: a method for preservation of water samples for eDNA analysis. BMC Res Notes. 2016;9:298. doi: 10.1186/s13104-016-2104-5 27278936 PMC4898389

[25] Pont D, Rocle M, Valentini A, Civade R, Jean P, Maire A, et al. Environmental DNA reveals quantitative patterns of fish biodiversity in large rivers despite its downstream transportation. Sci Rep. 2018;8(1):10361. doi: 10.1038/s41598-018-28424-8 29991759 PMC6039509

[26] Seymour M, Durance I, Cosby BJ, Ransom-Jones E, Deiner K, Ormerod SJ, et al. Acidity promotes degradation of multi-species environmental DNA in lotic mesocosms. Commun Biol. 2018;1:4. doi: 10.1038/s42003-017-0005-3 30271891 PMC6123786

[27] Coutant O, Richard-Hansen C, de Thoisy B, Decotte J-B, Valentini A, Dejean T, et al. Amazonian mammal monitoring using aquatic environmental DNA. Mol Ecol Resour. 2021;21(6):1875–88. doi: 10.1111/1755-0998.13393 33787010

[28] Jane SF, Wilcox TM, McKelvey KS, Young MK, Schwartz MK, Lowe WH, et al. Distance, flow and PCR inhibition: eDNA dynamics in two headwater streams. Mol Ecol Resour. 2015;15(1):216–27. doi: 10.1111/1755-0998.12285 24890199

[29] Chin SC, Waldman J, Bednarski M, Camhi M, LaBelle J, Elizabeth Alter S. Relating American Eel Abundance to Environmental DNA Concentration in the Bronx River. North American Journal of Fisheries Management. 2021;41(4):1141–50. doi: 10.1002/nafm.10625

[30] Ríos-Villamizar E, Piedade M, Costa J, Adeney J, Junk W. Chemistry of different Amazonian water types for river classification: a preliminary review. Environmental Science. 2014;178:17–28.

[31] Cantanhede AM, Da Silva VMF, Farias IP, Hrbek T, Lazzarini SM, Alves-Gomes J. Phylogeography and population genetics of the endangered Amazonian manatee, Trichechus inunguis Natterer, 1883 (Mammalia, Sirenia). Mol Ecol. 2005;14(2):401–13. doi: 10.1111/j.1365-294X.2004.02413.x 15660933

[32] Oberacker P, Stepper P, Bond DM, Höhn S, Focken J, Meyer V, et al. Bio-On-Magnetic-Beads (BOMB): Open platform for high-throughput nucleic acid extraction and manipulation. PLoS Biol. 2019;17(1):e3000107. doi: 10.1371/journal.pbio.3000107 30629605 PMC6343928

[33] Martin M. Cutadapt removes adapter sequences from high-throughput sequencing reads. EMBnet Journal. 2011;17(1):200.

[34] Rognes T, Flouri T, Nichols B, Quince C, Mahé F. VSEARCH: a versatile open source tool for metagenomics. PeerJ. 2016;4:e2584. doi: 10.7717/peerj.2584 27781170 PMC5075697

[35] Mahé F, Rognes T, Quince C, de Vargas C, Dunthorn M. Swarm: robust and fast clustering method for amplicon-based studies. PeerJ. 2014;2:e593. doi: 10.7717/peerj.593 25276506 PMC4178461

[36] da Silva V, D’Affonseca Neto J, Rodrigues Z. Concepção e nascimento do primeiro filhote de peixe-boi da Amazônia em cativeiro. Latin American Journal of Aquatic Mammals. 57.

[37] Domit C, Azevedo A, Meirelles A, Souza D, Attademo F, Silva F, et al. Trichechus inunguis: sistema de avaliação do risco de extinção da biodiversidade. ICMBio. 2023.

[38] Junk W, Piedade M, Wittman F, Schöngart J, Parolin P. Amazonian floodplain forests: ecophysiology, biodiversity and sustainable management. Springer Science and Business Media. 2011.

[39] Junk W, Piedade M. An introduction to South American wetland forests: Distribution, definitions and general characterization. Amazonian Floodplain Forests: Ecophysiology, Biodiversity, and Sustainable Management. 2010:3–25.

[40] Strickler KM, Fremier AK, Goldberg CS. Quantifying effects of UV-B, temperature, and pH on eDNA degradation in aquatic microcosms. Biological Conservation. 2015;183:85–92. doi: 10.1016/j.biocon.2014.11.038

[41] Deiner K, Fronhofer EA, Mächler E, Walser J-C, Altermatt F. Environmental DNA reveals that rivers are conveyer belts of biodiversity information. Nat Commun. 2016;7:12544. doi: 10.1038/ncomms12544 27572523 PMC5013555

[42] Da Silva VMF, César Weber Rosas F, D’Affonseca Neto JA, Feldberg E, Oliveira Franco-de-Sá JF, Lazzarini SM, et al. Oral cleft in an Amazonian manatee (Trichechus inunguis) (Mammalia, Sirenia). Lat Am J Aquat Mamm. 2024. doi: 10.5597/lajam00320

[43] Taberlet P, Bonin A, Zinger L, Coissac E. Environmental DNA: For biodiversity research and monitoring. Oxford Academic. 2018.

[44] Curtis AN, Tiemann JS, Douglass SA, Davis MA, Larson ER. High stream flows dilute environmental DNA (eDNA) concentrations and reduce detectability. Diversity and Distributions. 2020;27(10):1918–31. doi: 10.1111/ddi.13196

[45] Tsuji S, Ushio M, Sakurai S, Minamoto T, Yamanaka H. Water temperature-dependent degradation of environmental DNA and its relation to bacterial abundance. PLoS One. 2017;12(4):e0176608. doi: 10.1371/journal.pone.0176608 28448613 PMC5407774

[46] Turner CR, Uy KL, Everhart RC. Fish environmental DNA is more concentrated in aquatic sediments than surface water. Biological Conservation. 2015;183:93–102. doi: 10.1016/j.biocon.2014.11.017

[47] Seymour M. Rapid progression and future of environmental DNA research. Commun Biol. 2019;2:80. doi: 10.1038/s42003-019-0330-9 30820475 PMC6393415

[48] Sales NG, Wangensteen OS, Carvalho DC, Mariani S. Influence of preservation methods, sample medium and sampling time on eDNA recovery in a neotropical river. Environmental DNA. 2019;1(2). doi: 10.1002/edn3.14

