# Supplementary material for: Survey of vulnerable Amazonian manatees using environmental DNA (eDNA): A method for survey in remote field settings
Source: PLoS One. 2026 Feb 4;21(2):e0339410. doi: 10.1371/journal.pone.0339410 (PMC12872014; doi:10.1371/journal.pone.0339410)
Supplement: S1 Table — Codes starting with “Q” indicate unique Illumina primers for each sample, 1–90, with negative controls, N, throughout the 96-well plate. These primer pairs uniquely identify each sample so they can all be combined during library construction and subsequent steps. (PDF) [file pone.0339410.s001.pdf]

**Plate**		**1**	**2**	**3**	**4**	**5**	**6**	**7**	**8**	**9**	**10**	**11**	**12**
		**Q7027**	**Q7028**	**Q7029**	**Q7030**	**Q7035**	**Q7036**	**Q7039**	**Q7040**	**Q7041**	**Q7042**	**Q7047**	**Q7048**
**A**	**Q5013**	1	2	3	4	5	N1	6	7	8	9	10	11
**B**	**Q5014**	12	13	14	15	16	17	18	19	20	21	22	23
**C**	**Q5017**	24	25	26	27	28	29	30	N2	31	32	33	34
**D**	**Q5018**	35	36	37	38	39	40	41	42	43	44	45	46
**E**	**Q5025**	47	48	9	50	51	52	53	54	55	56	57	58
**F**	**Q5026**	59	60	N3	61	62	63	64	65	66	67	68	69
**G**	**Q5027**	70	71	72	73	74	75	76	77	N4	78	79	80
**H**	**Q5028**	81	82	83	84	85	86	87	88	89	90	N5	
